# Decline in older adults’ daily mobility during the COVID-19 pandemic: the role of individual and built environment factors

**DOI:** 10.1186/s12889-022-14780-8

**Published:** 2022-12-12

**Authors:** Eun Yeong Choe, Yao Du, Guibo Sun

**Affiliations:** Urban Analytics and Interventions Research Lab, Department of Urban Planning and Design, The University of Hong Kong, Hong Kong SAR, China

**Keywords:** Active travel, Public transport use, Travel behaviour, Post-pandemic, Hong Kong

## Abstract

**Background:**

Extensive research has shown that the COVID-19 pandemic dramatically impacted the daily mobility of older adults. However, very little attention has been paid to the role of individual and built environmental factors in decline in older adults’ daily mobility during the pandemic.

**Methods:**

Based on a cohort survey of 741 older adults in Hong Kong, we conducted a one-way ANOVA to explore the differences in determinants (individual or environmental factors) of older adults’ daily mobility between before and during the COVID-19 pandemic. Further, multilevel linear regression was performed to examine how individual characteristics and built environment factors are associated with changes in older adults’ daily mobility during the pandemic.

**Results:**

Results show that the duration of active travel declined from 174.72 to 76.92 min per week, and that the public transport use frequency decreased from an average of 6.14 to 3.96 trips per week during the COVID-19 pandemic (before the rollout of vaccination programme). We also found residential density (*p* < 0.05) and the number of bus stop was negatively associated with the decline in their active travel (*p* < 0.01), while a higher destination mix was associated with more significant decrease in active travel (*p* < 0.01). A higher availability of recreational facilities in neighbourhoods was associated with a greater decrease in public transport use (*p* < 0.05). In addition, those who were older or having depressive symptoms, which are considered a vulnerable group, were negatively associated with decrease in their mobility (*p* < 0.001).

**Conclusions:**

Maintaining mobility and social interactions are crucial for older adults’ health during the COVID-19 pandemic. This study found that individual and environmental factors differentially affected older adults’ active travel and public transport use during the pandemic. Our findings contribute to understanding the COVID-19 impact on daily mobility in older adults and support more effective active travel promotion policies in the post-pandemic future.

.

## Introduction

Since the COVID-19 pandemic started, there have been unprecedented changes in people’s daily mobility, physical activity and travel behaviour [1]. As older people over the age of 65 are more vulnerable to viruses, the current pandemic has forced them to isolate and keep a distance from others. In addition, opportunities to participate in most activities were cut down because restaurants, community centres and recreational facilities were closed, and cultural and religious events were cancelled. Consequently, older adults’ mobility was remarkably reduced by local restrictions, such as social distancing rules and temporary public transport service suspensions [2]. Moreover, the mobility limitation may leave older adults more vulnerable to loneliness and social isolation, which can affect their health and well-being [3].

A few studies have examined the effects of COVID-19 on older adults’ daily mobility including walking for transport and public transport use. Rantanen et al. showed that life-space mobility in older adults declined noticeably compared to the same quarter in 2018 [4]. Shaer and Haghshenas found that in the post-outbreak the average trip frequency per week declined from 10.5 to 3.7, and the average walking duration per week decreased from 59 to 29 min during the COVID-19 pandemic [5]. The study also reported that older adults did not use public transport to avoid crowds in the outbreak. Similarly, Suzuki et al. also found that the changes in older adults’ physical activity during the pandemic were related to the decreased transport-related activity [6]. A study in Korea showed the decline in public transport use caused by the sharp increase of COVID-19 infections was more noticeable among older people (65 years and over) than people aged between 20 and 64. The older adults reduced their metro use by 42% in a higher COVID-19 infection period [7]. However, there is still very little understanding of changes in older adults’ daily mobility and how they shape their mobility in reaction to COVID-19.

Individual characteristics significantly affect daily mobility among older people, such as gender, age, monthly income, and educational level. A study found that age was negatively related to older adults’ daily mobility [8]. More specifically, the older people’s walking trips increased with age, but their public transport use declined. They also showed males had more trips for work and recreational purposes than females; while females had more trips for shopping than males. Similarly, Legendre et al. showed that retired older women used public transport more frequently than men, and older women adopted public transport mostly for living necessities [9]. Monthly income and educational level are positively associated with the older adults’ trip-making and distance travelled [10, 11]. Kim’s study cited several studies in which it was found that older adults’ mobility is closely associated with income level, and that poor older people tend to be less active in non-home activities, particularly leisure activities [11]. Similarly, people with higher education levels tend to participate in social and community activities more than those with lower educational levels. In addition, several studies have examined the relationship between mental health and daily mobility. Dirik et al. showed that mental impairment was negatively associated with daily living activities and mobility among older adults [12]. Chiatti et al. reported that older people with a higher level of mental health status walked more than 500 m at least once a week and used public transport more frequently [13]. In a report by Posner and colleagues, the participants with mental health conditions mentioned that anxiety, depression or low mood, and stress negatively affected their daily mobility [14]. The most frequently reported problems were avoiding travel, lack of concentration, and unsafe or impaired travel behaviour. The relationship between mental health and daily mobility is complex: individuals’ mental health conditions can influence their ability to travel and personal experience. At the same time, travelling can cause stress that may worsen mental health difficulties [15]. For example, a person might choose to travel by bus rather than by metro because they have an irrational fear of confined spaces, and stress/anxiety can be caused by overcrowding and the complexity of information at bus stops, which may affect their mental health.

Several environmental variables have been used to examine factors that may affect older adults’ daily mobility. A meta-analysis study found positive associations with older adults’ walking for transport were shown for residential density, walkability, street connectivity, land use mix, and access to several types of destinations/services [16]. Similarly, a study also observed that walking in minutes per week was associated with residential density. The higher or better the density, street connectivity, aesthetics, traffic safety, proximity of destinations, the number of destinations within 20-min walk, and proximity of a bus stop, the higher the chance of walking for transport [17]. Neighbourhood green space is another significant predictor of active travel in older adults. Cheng et al. showed that the distance to the nearest park and square was significantly related to longer duration of active travel, suggesting that land use mix influenced the active travel pattern [18]. Moreover, Shaer et al. studied the impacts of COVID-19 on older adults’ active transport mode choice, including walking and cycling [19]. They found that quality of walking and cycling routes, intersections safety, greenery and public transport accessibility had positive effects on active mode choice of older adults. In terms of public transport use, both perceived and actual walking distance to bus stops/transit stations can influence on ridership for older adults [20]. Ning et al. found that the number of schools, hospitals, supermarkets, squares, parks, and scenic spots near metro stations significantly increases the proportion of older adults’ metro usage [21]. Further, residential density [20, 22], street connectivity [20], destination accessibility, and aesthetic and safety of pedestrian environment [23] affected the older adults’ public transport use. A study showed that public transportation accessibility and pedestrian environments influenced daily mobility in older people [24]. Similarly, Zhang et al. showed living in a neighbourhood with a high level of public transport service, and green spaces along walking routes connecting home and bus stops/transit stations is strongly related to more public transport trips of older people [25]. However, the association of environmental factors on older adults’ daily mobility differs by the specific environmental characteristics and travel modes. Yang and colleagues found that some neighbourhood features such as walkability affected both active travel and public transport use in the same direction, but other features were associated with the two behaviours in opposite directions [26]. For example, as the distance to the nearest park increased, public transport use was less likely but active travel more likely. In addition, higher levels of street connectivity were associated with higher odds of public transport use but lower odds of engaging in active travel. Thus, the association of the built environment is not always the same for active travel and public transport use.

There have been no studies which compare differences in individual and environmental factors affecting daily mobility before and during COVID-19. Although many studies have examined which individual and environmental factors affect older adults’ daily mobility, most of the existing studies conducted before the pandemic and showed inconsistent results of these factors. Therefore, this study investigates the changes in older adults’ daily mobility during the pandemic and explores how these are associated with individual characteristics and built environment factors.

## Materials and methods

### Metro and elderly health in Hong Kong study

We used longitudinal data from the Metro and Elderly Health in Hong Kong study [27]. The natural experimental study examines the impacts of a new metro line on older adults’ health and well-being. Details on the research design have been described in the published protocol [27]. The new metro line went into operation in June 2021. Both baseline and telephone surveys were conducted before the opening of the new metro line. Participants who were 65 years of age or older were recruited from neighbourhood elderly centres located in urban areas of Hong Kong. Baseline data were collected in 2019 before the COVID-19 pandemic. Trained interviewers conducted a face-to-face interview with each participant using a set of questionnaires to measure their public transport use behaviours, physical activity, perceived neighbourhood walkability and broader health outcomes. For the follow-up survey in September 2020 during the pandemic, we conducted a telephone-based survey of COVID-19’s potential impact on daily mobility of the cohort of participants. The follow-up survey dataset was matched with the baseline survey dataset using a unique identifier. After removing entries with missing identifiers, the baseline dataset was 826 participants, and the follow-up was 741. The matched dataset of 741 participants was used for analyses. All participants provided written informed consent with ethical approval from Research Ethics Committee of the University of Hong Kong.

### Variables

#### Daily mobility

The participants’ daily mobility was assessed with (1) duration of active travel, and (2) frequency of public transport use. Active travel was defined as walking undertaken for the purpose of going to/from places. We applied the Chinese version of the International Physical Activity Questionnaire - Short Form (IPAQ-SC) to calculate the participants’ weekly minutes of walking for transport in the past seven days. The total score (min) was treated as a continuous variable. Public transport use was defined as using shared vehicles (e.g., metro and bus) for daily mobility. The frequency of public transport use was obtained based on a set of questions about their weekly trip by public transport. At the baseline survey, the questions about the number of public transport use were asked (i.e., *How many times do you use metro/bus every week?*). Later, follow-up questions included how much they changed the frequency in each public transport use during the pandemic (e.g., *Did you change the number of metro/bus use after the spread of COVID-19? If yes, how many times did you increase or reduce in your weekly trip?*). The total score (time) was treated as a continuous variable.

#### Individual factors

Individual factors included gender (male, female), age (65–69, 70–79, over 80 years old), monthly income (below and above HK$3,500), education level (primary or below, secondary or above), retirement status, marital status (single, married), prevalence of chronic diseases (yes, no), an impact on mobility (low, moderate and significant) and depressive symptoms. Depressive symptoms as a common mental disorder were measured by Patient Health Questionnaire-9 (PHQ-9) [28], which scores each of the nine individual questions as “0” (not at all) to “3” (nearly every day). The PHQ-9 total score of 0–4 points indicates minimal depression, 5–14 points for mild to moderate depression, and 15 or more points for severe depression. According to their PHQ-9 score, we categorised people into two groups as minimal (below 5 points) and mild to moderate depression (above 5 points); this study did not include people with severe depression.

#### Built environment factors

We used a 400-m pedestrian network buffer from participants’ residential addresses to create six built environment factors for older adults. Residential density was extracted from population census data in 2016. The block census tract unit was defined by streets, containing the number of family members living within this unit. We calculated the population number based on covered blocks of the network buffers. Street connectivity was calculated as the link-node ratio which is equal to the number of links divided by the number of nodes within study area [29]. Destination mix was calculated by an entropy index based on fourteen destination categories (e.g., commercial, industrial, recreational facilities, healthcare services, government and institutions, schools and restaurants etc.), ranging from 0 (homogeneous land use) to 1 (equal mix of land use) [29]. The number of bus stops was measured, with a higher number indicating higher accessibility. Regarding the destination domain, the number of services was measured, with a higher number indicating higher availability. Recreational facilities include tourist attractions, sports and leisure centres, and public green spaces. Restaurants include local and international casual dining, fast-food restaurants and coffee shops. These destinations were previously identified as relating to older adults’ daily needs in Hong Kong [30].

A geographic information system (ArcGIS v10.7.1, Esri) was used to create built environment variables.

### Data analysis

We conducted a descriptive statistic to analyse all variables. A paired samples t-test was also applied to determine the changes in older adults’ daily mobility, both active travel and public transport use, during the pandemic. Further, a one-way ANOVA was conducted to examine the differences in determinants (individual or built environment factors) of older adults’ daily mobility between before and during the COVID-19 pandemic.

Two levels of built environment factors (i.e., individual and neighbourhood-levels) were used in multilevel linear regression. The multilevel linear regression was performed in three stages: (1) a null, random-effects model (Model 0) was estimated for the changes in active travel and public transport use, respectively. The null model provided the estimates of variance at both individual and environmental factors before any characteristics at these two levels were added; (2) individual factors were added to the models in sequence (Models 1); and (3) environmental factors are added to Model 2. We standardised all the variables before including them in the regression.

All analyses were carried out using Stata (v. 17, StataCorp).

## Results

### Descriptive results

Descriptive statistics of the study sample are presented in Table 1. The participants were 63.20% female, with a mean age of 75.80 years (SD = 6.97, range 65–92). About 78% of the participants were of primary education level or below. The sample comprised of 43.99% single and 56.01% married. The majority of the participants were retired and had at least one chronic disease, such as heart disease, diabetes or hypertension. The participants’ mean scores on PHQ-9 were 3.52, with minimal (69.20%) and mild-moderate depressive symptoms (30.80%).

We found a decrease in active travel and public transport use among older adults during the pandemic. The average walking duration per week declined from 174.72 to 76.92 min; *t* (740) = 26.71, *p* < 0.001, and the average public transport use frequency per week decreased from 6.14 to 3.96 times during the COVID-19 pandemic; *t* (740) = 32.48, *p* < 0.001.

**Table 1:** Summary of descriptive statistics

	Mean (SD) / N (%)
**Individual variables**	
Gender	
*Male*	273 (36.80)
*Female*	468 (63.20)
Age	
*65–69*	129 (17.40)
*70–79*	383 (51.70)
*80* +	229 (30.90)
Monthly income	
≤ *HKD 3500*	375 (50.60)
> *HKD 3500*	366 (49.40)
Education levels	
*Primary or below*	578 (78.10)
*Secondary or above*	162 (21.89)
Retirement status (Retired)	730 (98.52)
Marital status	
* Single*	326 (43.99)
*Married*	415 (56.01)
Prevalence of chronic diseases	614 (82.90)
Impact on mobility	
*Low*	552 (74.49)
*M****oderate**** and significant*	189 (25.51)
Depressive symptoms (range = 0–27)	
*Minimal*	513 (69.20)
*Mild to moderate*	228 (30.80)
**Built environment variables**	
Residential density	11.5 (0.86)
Street connectivity	1.6 (0.10)
Number of bus stops	13.7 (9.20)
Number of recreational facilities	10.1 (9.30)
Number of restaurants	39.9 (41.20)
Destination mix	0.6 (0.20)
**Outcome variables**	
Duration of active travel (before COVID-19)	174.72 (85.26)
Duration of active travel (during COVID-19)	76.95 (53.40)
Frequency of public transport use (before COVID-19)	6.14 (2.64)
Frequency of public transport use (during COVID-19)	3.96 (2.25)
Change in duration of active travel	97.77 (69.27)
Change in frequency of public transport use	2.18 (2.43)

### The effects of individual and environmental factors on older adults’ daily mobility before and during the COVID-19 pandemic

Table 2 shows the results of one-way ANOVA analysis and whether there were significant differences of individual and built environment variables between groups. Before COVID-19, there were significant differences between gender, age, monthly income and depressive symptoms in older adults’ active travel (*p* < 0.001 for gender, age and depressive symptoms; *p* < 0.01 for monthly income). Among built environment variables, there were significant differences between the number of bus stops, the number of restaurants and destination mix in older adults’ active travel (*p* < 0.001 for the number of bus stops; *p* < 0.01 for the number of restaurants and destination mix). However, during the COVID-19 pandemic, there were no significant differences between the individual factors in older adults’ active travel. Significant differences were found between residential density, the number of recreational facilities and restaurants, and destination mix in environmental factors in older adults’ active travel (*p* < 0.05).

In regards to public transport use, before COVID-19 there were significant differences between age, monthly income, education level, the presence of chronic diseases and depressive symptoms in older adults’ public transport use (*p* < 0.001 for education level, the presence of chronic diseases and depressive symptoms; *p* < 0.05 for age and monthly income). However, no significant differences of environmental factors in public transport use among older people were found. During COVID-19, there were still significant differences between education level and depressive symptoms in older adults’ public transport use (*p* < 0.001 for depressive symptoms; *p* < 0.05 for education level), and marital status became significant (*p* < 0.001). A significant difference was found between the number of recreational facilities and restaurants in older adults’ public transport use (*p* < 0.05).

**Table 2:** Differences in individual and built environment variables for daily mobility before and during COVID-19

	Active travel (min)				Public transport use (time)			
	Before COVID-19 (*n* = 826)		During COVID-19 (*n* = 741)		Before COVID-19 (*n* = 826)		During COVID-19 (*n* = 741)	
	Mean (SD)	F	Mean (SD)	F	Mean (SD)	F	Mean (SD)	F
**Individual variables**								
Gender								
*Male*	159.95 (80.65)	14.78***	80.13 (53.06)	1.53	6.02 (2.59)	0.93	3.90 (2.08)	0.24
*Female*	183.74 (88.52)		75.10 (53.57)		6.22 (2.66)		3.98 (2.34)	
Age								
*65–69*	191.16 (89.15)	9.21***	73.64 (52.70)	0.88	6.15 (2.75)	3.91*	4.01 (2.34)	1.99
*70–79*	180.33 (84.91)		75.85 (53.27)		6.37 (2.58)		4.09 (2.20)	
*80* +	156.88 (84.58)		80.65 (54.03)		5.76 (2.63)		3.72 (2.26)	
Monthly income								
≤ *HKD 3500*	166.40 (85.67)	8.83**	76.71 (54.54)	0.02	5.95 (2.65)	4.06*	3.95 (2.31)	0.01
> *HKD 3500*	184.20 (86.40)		77.20 (52.28)		6.34 (2.62)		3.97 (2.19)	
Education levels								
*Primary or below*	173.58 (87.23)	0.96	76.74 (52.59)	0.06	5.97 (2.69)	11.99***	3.78 (2.23)	5.24*
*Secondary or above*	180.73 (83.46)		77.93 (56.44)		6.77 (2.36)		4.56 (2.22)	
Retirement status								
*Yes*	174.40 (85.49)	0.66	76.71 (53.34)	0.97	6.13 (2.63)	1.18	3.95 (2.25)	0.54
*No*	195.45 (68.32)		92.73 (57.64)		7 (2.86)		4.45 (1.86)	
Marital status								
*Single*	174.06 (87.43)	0.03	76.92 (53.66)	0.01	6.01 (2.61)	1.60	3.73 (2.18)	5.88**
*Married*	175.23 (83.62)		76.97 (53.26)		6.25 (2.66)		4.13 (2.28)	
Presence of chronic diseases								
*Yes*	175.80 (87.56)	0.33	77.27 (54.18)	0.13	5.98 (2.65)	13.80***	3.89 (2.23)	2.92
*No*	171.21 (80.89)		75.39 (49.63)		6.93 (2.46)		4.27 (2.29)	
Impact on mobility								
*Minimal*	177.49 (84.31)	2.29	76.92 (53.75)	0.01	6.12 (2.62)	0.14	3.97 (2.24)	0.02
*Mild to moderate*	166.62 (87.72)		77.03 (52.51)		6.21 (2.68)		3.94 (2.27)	
Depressive symptoms								
*Minimal*	185.62 (85.12)	28.93***	76.70 (54.52)	0.03	7.22 (2.17)	448.39***	4.72 (2.07)	255.93***
*Mild to moderate*	151.14 (84.74)		77.50 (50.89)		3.71 (1.87)		2.25 (1.59)	
**Built environment variables**								
Residential density								
≤ *11.5*	171.60 (87.63)	0.43	72.89 (51.17)	2.78*	6.03 (2.70)	0.95	3.97 (2.20)	0.03
> *11.5*	175.71 (84.73)		79.69 (54.51)		6.22 (2.62)		3.94 (2.27)	
Street connectivity								
≤ *1.6*	174.11 (85.72)	0.21	76.37 (55.61)	0.22	6.05 (2.64)	2.63	3.94 (2.23)	0.08
> 1.6	177.15 (88.23)		78.42 (47.52)		6.39 (2.61)		3.99 (2.30)	
Number of bus stops								
≤ *13.9*	184.45 (89.07)	13.88***	74.88 (51.49)	1.38	6.07 (2.62)	0.51	4.01 (2.27)	0.60
> 13.9	162.07 (80.26)		79.55 (55.32)		6.22 (2.67)		3.88 (2.21)	
Number of recreational facilities								
≤ *10*	177.18 (89.49)	1.78	73.46 (52.76)	5.63*	6.13 (2.76)	0.05	4.08 (2.31)	4.07*
> 10	168.85 (78.99)		83.19 (53.76)		6.17 (2.43)		3.73 (2.11)	
Number of restaurants								
≤ *41*	179.25 (90.49)	4.30*	73.62 (51.70)	4.43*	6.24 (2.72)	1.29	4.06 (2.22)	3.69*
> 41	166.53 (77.92)		82.13 (55.32)		6.01 (2.53)		3.79 (2.28)	
Destination mix								
≤ *0.6*	163.48 (81.74)	4.69*	71.24 (52.04)	6.74*	6.13 (2.59)	0.01	3.85 (2.33)	0.63
> 0.6	178.10 (86.99)		85.40 (54.97)		6.15 (2.67)		3.99 (2.21)	

^*^p < 0.05; **p < 0.01; ***p < 0.001

### Individual and environmental factors affecting decline in older adults’ daily mobility during the COVID-19 pandemic

Table 3 presents the results from multilevel linear regression models for impacts of individual and environmental factors on decline in older adults’ daily mobility, both active travel and public transport use. Model 0 shows that there was significant variation in decrease in older adults’ daily mobility (variance components for the intercept, *p* < 0.001), with around 2% and 3% of the total variation among the study sites. After the individual variables were added to the model, the variation increased by 1% for public transport use. Model 1 illustrates that those of older age and having depressive symptoms were negatively associated with decrease in active travel among older people (*p* < 0.001 for depressive symptoms; *p* < 0.01 for age), while females were associated with greater decrease in active travel (*p* < 0.001). For public transport use, those of higher income and having chronic diseases were associated with greater decreases (*p* < 0.001 for the presence of chronic diseases; *p* < 0.05 for monthly income), while those with depressive symptoms were negatively associated with decrease during the COVID-19 pandemic (*p* < 0.001). Model 2 further added built environment variables. The variation in both active travel and public transport use remains unchanged. The results showed that residential density (*p* < 0.05) and the number of bus stops were negatively associated with decreases in active travel (*p* < 0.01), while destination mix was associated with greater decrease in active travel (*p* < 0.01). For public transport use, a greater number of recreational facilities was associated with greater decrease among older people (*p* < 0.05). After adding environmental variables, the effects of four individual factors remained unchanged.

**Table 3:** Effects of the individual and built environment variables on decline in daily mobility during COVID-19; Coefficient (*β)* and 95% CIs for active travel and public transport use

	Model 0		Model 1		Model 2	
	Active travel	Public transport use	Active travel	Public transport use	Active travel	Public transport use
**Fixed effects**						
Intercept	0.02*** (0.01;0.11)	0.03*** (0.01;0.14)	0.14* (0.05; 0.47)	0.42** (0.18; 1.03)	0.11* (0.07; 0.49)	0.44 (-0.16; 1.04)
**Individual variables**						
Female			0.28*** (0.14; 0.43)	0.08 (-0.06; 0.22)	0.27*** (0.13; 0.42)	0.08 (-0.06; 0.22)
Age						
*65–69*						
*70–79*			-0.13 (-0.33; 0.07)	0.09 (-0.11; 0.28)	-0.14 (-0.34; 0.06)	0.08 (-0.12; 0.27)
*80* +			-0.33* (-0.55; -0.12)	-0.01 (-0.22; 0.21)	-0.35*** (-0.57; -0.14)	0.01 (-0.21; 0.22)
Monthly income *(*> *HKD 3500)*			0.12 (-0.01; 0.27)	0.14* (0.01; 0.28)	0.14* (-0.01; 0.28)	0.14* (0.01; 0.28)
Education level *(Secondary school or above)*			0.05 (-0.12; 0.23)	-0.05 (-0.23; 0.12)	0.06 (-0.11; 0.23)	-0.06 (-0.23; 0.12)
Retirement status (Retired)			0.13 (-0.45; 0.73)	-0.16 (-0.75; 0.42)	0.13 (-0.45; 0.72)	-0.18 (-0.76; 0.40)
Marital status (Married)			0.02 (-0.12; 0.17)	-0.10 (-0.25; 0.04)	0.01 (-0.13; 0.16)	-0.10 (-0.24; 0.04)
Presence of chronic diseases			0.04 (-0.14; 0.22)	-0.25** (-0.44; -0.07)	0.01 (-0.16; 0.21)	-0.26** (-0.44; -0.08)
Impact on mobility (Mild to moderate)			-0.10 (-0.26; 0.06)	0.08 (-0.08; 0.24)	-0.10 (-0.26; 0.06)	0.09 (-0.07; 0.25)
Depressive symptoms (Mild to moderate)			-0.32*** (-0.47; -0.17)	-0.53*** (-0.68; -0.38)	-0.28*** (-0.43; -0.13)	-0.53*** (-0.67; -0.38)
**Built environment variables**						
Residential density					-0.07* (-0.14; -0.01)	0.04 (-0.03; 0.11)
Street connectivity					0.01 (-0.07; 0.07)	0.02 (-0.04; 0.15)
Number of bus stops					-0.17** (-0.28; -0.06)	0.03 (-0.08; 0.03)
Number of recreational facilities					0.06 (-0.44; 0.15)	0.10* (0.01; 0.20)
Number of restaurants					0.01 (-0.09; 0.11)	-0.09 (-0.20; 0.02)
Destination mix					0.11** (0.03; 0.18)	-0.01 (-0.09; 0.06)
**Random variance component**						
Between-group variance	0.14 (0.07; 0.29)	0.19 (0.10; 0.35)	0.13 (0.06; 0.28)	0.18 (0.10; 0.34)	0.08 (0.02; 0.43)	0.14 (0.06; 0.31)
Within-group variance	0.99 (0.94; 1.04)	0.98 (0.93; 1.03)	0.95 (0.91; 1.01)	0.94 (0.89; 0.98)	0.95 (0.90; 0.99)	0.93 (0.89; 0.98)
ICC	0.02	0.03	0.02	0.04	0.02	0.03
AIC	2101.24	2093.76	2068.40	2042.37	2062.09	2047.15

^*^p < 0.05; **p < 0.01; ***p < 0.001

Model 0 (Unconditional model); Model 1 (+ individual variables); Model 2 (+ individual variables and environmental variables)

## Discussion

### Primary findings

This study is the first to explore the role of individual and built environment factors in the decline in older adults’ daily mobility during the COVID-19 pandemic. We found a noticeable decline in active travel and public transport use among older adults during the pandemic. One of the more significant findings from this study is that individual differences in older adults’ active travel became insignificant under pandemic conditions. Second, environmental factors were more influential predictors for active travel than for public transport use among older people. Last, more physically active older adults were more likely to decrease their daily mobility during the pandemic.

### Interpretations and implications

This study identified that individual differences in older adults’ active travel became not significant under pandemic conditions. Consistent with the literature [31, 32], our study also indicated that gender, age, monthly income and mental health conditions were significant predictors for both active travel and public transport use among older people before the pandemic. However, age and monthly income became non-significant factors for public transport use, and no individual factors affected active travel during COVID-19. After the outbreak, local and national restrictions have reduced public services and activities; it might affect older people’s daily mobility beyond their individual differences.

Second, built environment factors were more influential predictors for active travel than public transport use among older people. We found that the number of bus stops, the number of restaurants and destination mix significantly influenced active travel (particularly walking), while few environmental factors were associated with public transport use. This confirms the findings of previous studies about the impacts of neighbourhood environments on older adults’ active travel and activities, which indicated such relations in non-pandemic conditions, also during the pandemic [33, 34]. As older adults become less mobile, their active spaces may shrink over time to include only the immediate areas within walking distance of their homes [35]. Extensive research points to residential density, street and traffic conditions, and proximity to destinations and green spaces as the most likely factors influencing mobility in older adults in positive and negative directions [36, 37]. Especially under the disruptive situation like COVID-19, older people are more likely to be affected by the neighbourhood environment due to accelerated mobility decline. A similar study indicated that the availability of daily amenities, such as grocery shops, restaurants, sports and recreational facilities and healthcare services within a 15–20 min walking distance can maintain daily life flux for individuals coping with the lockdown restrictions during the pandemic [33].

In particular, the availability of recreational facilities became an important factor for both active travel and public transport use during the pandemic. These findings are in accord with previous studies suggesting that various recreational destinations were positively associated with older adults’ mobility [18]. Neighbourhoods with more recreational facilities, such as tourist attractions, sports and leisure centres and public green spaces, may offer older adults an attractive opportunity to travel [38]. In addition, a recent study showed that individuals’ participation in out-of-home activities was reduced by more than 50% during COVID-19, but recreational travel activities increased with a higher share of older adults after adjustment in out-of-home activities during the pandemic [39].

Further, our findings suggest that more physically active older adults were more likely to decrease their daily mobility during the pandemic. The results from multilevel linear regression found that those of older age and having mild to moderate depressive symptoms, which are considered to be particularly a vulnerable group, were associated with less decrease in their mobility. It implied the inactive older adults were still constrained in their travel options during COVID-19. In addition, the results also showed that those in higher destination mixed area and higher availability of recreation facilities had greater decrease in active travel. Several studies have indicated that the accessibility of transit stops and the availability of essential services in neighbourhoods strongly relate to individual travel behaviour. For example, the diversity of destinations in a neighbourhood was positively associated with average walking time among older people [40]. A study also found strong associations with older adults’ active travel were shown for residential density, land use mix, and access to several types of destinations/services [16]. In other words, those living in higher residential density and higher diversity of destinations/services are more likely to physically active. Due to voluntary (social isolation) and/or involuntary (lockdown) social distancing imposed by the pandemic, older adults’ active travel and physical activity, particularly in more active older people, have deteriorated during COVID-19.

### Limitations and strengths

This study has some limitations. One weakness in this study was that the results might reflect recall bias involving reports of active travelling duration and public transport use. Some survey respondents might underreport or overreport their trips. Another limitation was that social distancing restrictions could have affected built environment variables during the pandemic. During the study period, the government tightened social distancing measures such as temporarily closed venues and group gathering limits. Access to recreational facilities or essential services has been limited during the outbreak (before the rollout of vaccination programme); it might affect the measurement of recreational facilities or restaurants in this study. Finally, this study only addressed the situation in the early stages of the outbreak. Our findings showed daily mobility changes in response to a specific and unexpected event rather than long-term mobility patterns. It would be worthwhile to explore subsequent changes in mobility patterns with increasing pandemic dynamics in further research.

Notwithstanding these limitations, we have attempted to provide insight into whether individual and environmental factors affecting older adults’ daily mobility differ before and during the pandemic and if so, how work they are. This study certainly adds to our understanding of the impacts of COVID-19 on individuals’ mobility among older adults.

## Conclusion

Maintaining mobility and social interactions are crucial for older adults’ health during the COVID-19 pandemic. This study found that individual and environmental factors differentially affected older adults’ active travel and public transport use during the pandemic. We also found more physically active older adults were more likely to decrease their mobility in the critical condition of the pandemic. This study contributes to understanding the COVID-19 impact on daily mobility in older adults and supports more effective active travel promotion policies in the post-pandemic future.

## Data Availability

The data generated and analysed during the current study are available from the corresponding author on reasonable request.
